# Periodontal conditions in patients with Marfan syndrome – a multicenter case control study

**DOI:** 10.1186/1472-6831-13-59

**Published:** 2013-10-28

**Authors:** Ingmar Staufenbiel, Christian Hauschild, Bärbel Kahl-Nieke, Eva Vahle-Hinz, Yskert von Kodolitsch, Maike Berner, Oskar Bauss, Werner Geurtsen, Alexander Rahman

**Affiliations:** 1 Department of Conservative Dentistry, Periodontology and Preventive Dentistry, Hannover Medical School, Carl-Neuberg-Strasse 1, 30625, Hannover, Germany; 2 Department of Orthodontics, University Medical Center Hamburg-Eppendorf, Hamburg, Germany; 3 Center of Cardiology and Cardiovascular Surgery, University Medical Center Hamburg-Eppendorf, Hamburg, Germany; 4 Private Practice, Hannover, Germany

**Keywords:** Marfan syndrome, Periodontal conditions, Periodontitis, Inflammation signs, Attachment-loss, Dental conditions, Oral manifestations

## Abstract

**Background:**

Marfan syndrome (MFS) is a disorder of the connective tissues. Alterations of the elastic fibers may manifest in different tissues especially in the skeletal, cardiovascular and ocular system. Oral manifestations like orthodontic or skeletal anomalies and fragility of the temporomandibular joint have been well described by various authors. However, no data are available regarding a possible periodontal involvement of MFS. Hence, the aim of the present study was to investigate for the first time if MFS may increase the susceptibility to periodontitis.

**Methods:**

A comprehensive periodontal examination including documentation of probing pocket depth, gingival recession, clinical attachment level, and bleeding on probing was conducted in all patients. In addition, dental conditions were assessed by determining the Index for Decayed, Missing and Filled Teeth (DMFT) and a self-administered questionnaire was filled out by patients. For statistical analysis, the unpaired t-Test was applied (level of significance: p < 0.05). Both groups were matched concerning well known periodontal risk factors like age, gender and smoking habits.

**Results:**

82 participants, 51 patients with MFS (30 female and 21 male, mean age: 40.20 ± 15.35 years) and 31 sound controls (17 female and 14 male, mean age: 40.29 ± 13.94 years), were examined. All assessed periodontal and dental parameters were not significantly different between groups.

**Conclusions:**

Based on our data, patients with MFS did not reveal a higher prevalence of periodontitis compared to the control group. However, Marfan patients showed a tendency to more inflammation signs, which can be explained by the crowded teeth. Therefore, a regular professional cleaning of the teeth is recommendable (i.e., 6 months intervals) in order to reduce the bacterial biofilm in the oral cavity and thus resulting in a decreased risk of systemic diseases, specifically endocarditis.

## Background

**Figure 1:**
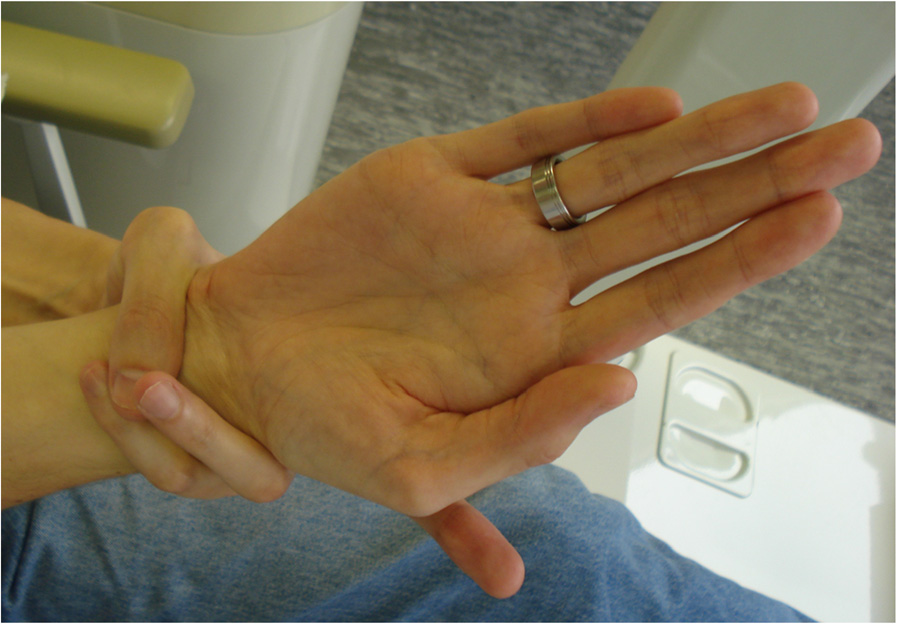
**A positive wrist sign in a patient with Marfan syndrome.** In case of a positive wrist sign the thumb and little finger overlap, when grasping the wrist of the opposite hand.

**Figure 2:**
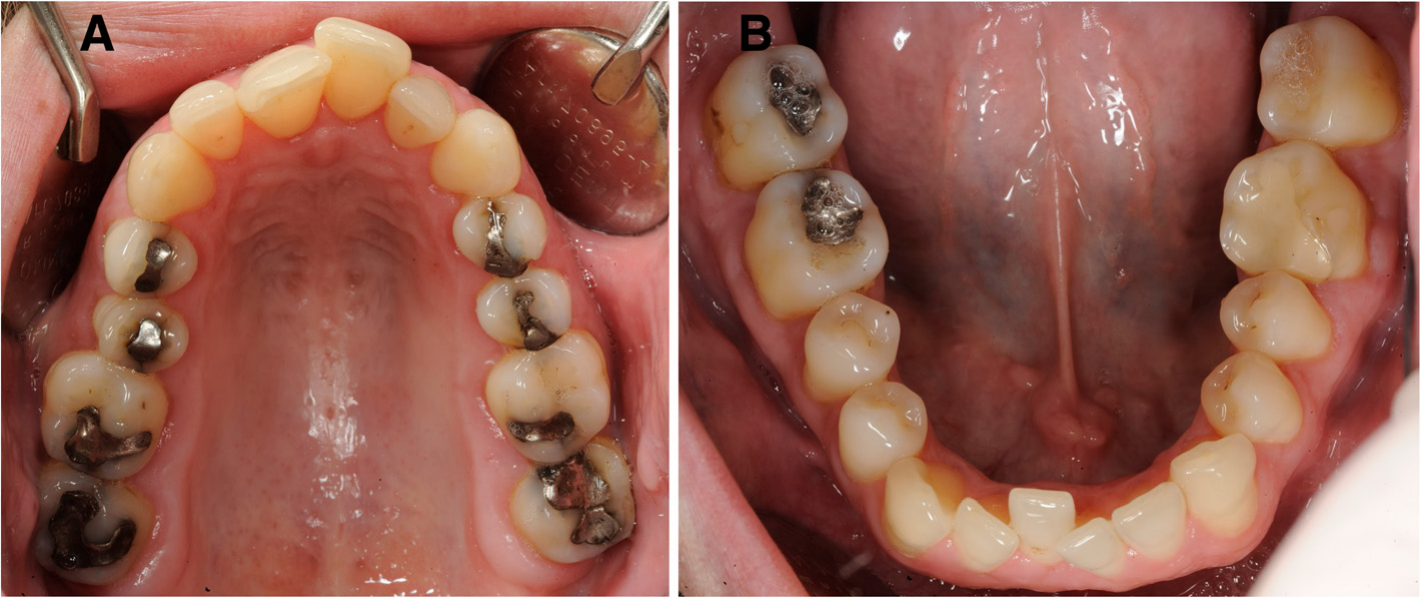
**Characteristic oral findings (high palate and crowded teeth) without periodontal abnormalities. A** Occlusal view of the upper jaw in a patient with Marfan syndrome. **B** Occlusal view of the lower jaw in a patient with Marfan syndrome.

Marfan syndrome (MFS), firstly described in 1896 by Antoine-Bernard Marfan [1], is an autosomal dominant disorder affecting the connective tissues. The prevalence of Marfan syndrome is 1 per 5,000 [2]. Mutations in the fibrillin-1 gene on chromosome 15 are responsible for alterations of the glycoprotein fibrillin-1, a major component of the 10–12 nm microfibrils present in the connective tissue matrices [2,3]. These microfibrils, together with elastin form the elastic fibers found in various tissues, including the suspensory ligament of the lens, skeletal system, lungs, blood vessel walls, and the skin [4]. Thus, MFS is a multisystemic disease where localization and degree of symptoms are individually different. Some clinical manifestations are characteristic for MFS, e.g., a positive wrist sign (Figure 1), tall stature, aortic dissection, mitral valve prolapse, and ectopia lentis [5]. Cardiovascular complications, especially aortic dissection or ruptures, are the major cause of morbidity and mortality [5,6]. Although genetic tests are available, the diagnostic criteria of the current Ghent nosology still require clinical manifestations for final diagnosis [7]. In addition to the aforementioned multisystemic manifestations, MFS exhibits characteristic oral features including maxillary protrusion, high palate (Figure 2A), crowded teeth (Figure 2B), and fragility of the temporomandibular joint [8,9].

Only scarce data are available about periodontal conditions in patients with MFS. Except for *De Coster et al.*[10] all publications are case reports [11-13]. Periodontal manifestations, a higher prevalence of gingivitis and/or periodontitis in patients with MFS may be considered because of the high concentration of elastic fibers in the periodontal ligament. Gingivitis and periodontitis are chronic inflammatory diseases of the tooth supporting tissues (periodontium) caused by bacteria of the oral cavity that accumulate on the teeth thus forming a biofilm [14]. The prevalence of gingivitis in the western populations is almost 75%. Approximately 30% of adults in the United States reveal moderate forms of periodontitis and 10% of the US-population is affected by severe forms of this disease [15]. Bacterial biofilm is the primary risk factor of gingivitis and periodontitis. But there are secondary genetic and so called alterable (patient-specific) risk factors as well, which affect host specific immune response and metabolism of the tooth supporting tissues [16]. These risk factors can be summarized as ‘susceptibility factors’ to periodontitis.

Therefore, the objective of the present case control study was to analyze if the mutated elastic fibers and the extracellular matrix metabolism and composition changes in the periodontal ligament in patients suffering from MFS may increase the susceptibility to periodontitis: The question which was to be answered was do patients with MFS reveal significantly more periodontal damage and inflammation signs? The hypothesis that was set forth was that patients with MFS have a higher prevalence of periodontitis.

## Methods

In accordance with the Declaration of Helsinki, the present study was approved by the Ethical Committee of Hannover Medical School (No. 5113) and the Ethical Committee of the Medical Association of Hamburg (No. MC-120/10). The study was designed as a multicenter case control study.

### Patients

A total of 82 participants, 51 patients with clinically and genetically verified MFS and 31 control persons without systemic diseases were included in the study. Most patients with MFS were recruited by the interdisciplinary Marfan consultation group of the University Medical Center, Hamburg-Eppendorf. In addition, few patients were recruited by the Institute of Human Genetics of Hannover Medical School or via the official website of the German Marfan-Hilfe e.V.

Exclusion criteria for both groups were: (1) systemic diseases that may negatively influence periodontal conditions (e.g. diabetes mellitus or infectious diseases like HIV-Infection), (2) pregnancy or breastfeeding, and (3) history of drug abuse.

Both groups were matched concerning well known periodontal risk factors like age, gender, and smoking habits.

Detailed instructions were given to the patients including an information brochure explaining the study design. All subjects signed an informed consent form. The investigations were conducted between October 2010 and November 2011.

### Questionnaire

A questionnaire was handed out in the Marfan group to ask about systemic manifestations of MFS and Marfan specific surgical interventions. In addition, patients were asked about a previous orthodontic treatment, frequency of dentist visit, and disorders of the temporomandibular joint.

### Periodontal examination

All patients underwent a comprehensive periodontal examination including probing pocket depth (PPD), gingival recession (GR), clinical attachment level (CAL), and bleeding on probing (BOP) assessed at six sites per tooth (disto-buccal, buccal, mesio-buccal, mesio-oral, oral, disto-oral). All measurements were carried out by two calibrated experienced investigators using a pressure-calibrated probe (TPS probe, Vivacare, Schaan, Liechtenstein). The tip of the probe had a diameter of 0.5 mm and the probing force was set at 20 g. Depending on PPDs the Periodontal Screening Index (PSI) was determined [17]. The degree of tooth mobility [18] and for multi-rooted teeth the degree of furcation involvement [19] was documented. Mean number and highest degree of tooth mobility and furcation involvement as well were calculated.

### Dental examination

A dental examination was performed in all patients including fillings, caries, crowns, and missing teeth. All decayed, missing, filled teeth and surfaces were summed up and the Index for ‘Decayed’ , ‘Missing’ and ‘Filled Teeth’ (DMF-T) as well as ‘Surfaces’ (DMF-S), was calculated [20].

### Statistical analysis

The recorded data were documented and analysed by the data procession program SPSS 19.0 for Windows (SPSS, Chicago, IL, USA). Each individual subject counted as a statistical unit of all tests. Mean and range values were calculated for all parameters. The unpaired t-Test was applied to determine significant differences between the groups. For all tests a p-value of p ≤ 0.05 was considered to be statistically significant. The questionnaire was analysed descriptively.

## Results

A total of 82 patients, 51 patients with MFS (30 female and 21 male, mean age: 40.20 ± 15.35 years) and 31 controls (17 female and 14 male, mean age: 40.29 ± 13.94 years), were examined.

### Questionnaire

Regarding systemic manifestations of MFS, 88% of the Marfan patients had an involvement of the cardiovascular system, 72% of the skeletal system, and 62% of the optical system. 74% of the Marfan patients had previously undergone surgical interventions, mostly due to cardiovascular diseases.

With respect to the frequency of dentist visit, 52.9% of the Marfan patients attended dental examination twice a year, whereas 33.3% visited a dental office only annually. The remaining patients were examined three times a year (7.8%) or irregularly (5.9%).

39.2% of the Marfan patients were affected by disorders of the temporomandibular joint and 62% had a previous orthodontic treatment.

### Periodontal examination

Mean and range values of all periodontal parameters (PPD, GR, CAL, BOP, PSI, degree of tooth mobility, degree of furcation involvement) are presented in Table 1.

**Table 1:** Mean and range values of all assessed clinical periodontal parameters and comparison of mean values (unpaired t-Test)

**Clinical parameter**	**Marfan**	**Control**
	**group (n = 51)**	**group (n = 31)**
Probing pocket depth (in mm)	2.33 ± 0.43*	2.39 ± 0.40
Gingival recession (in mm)	0.33 ± 0.34*	0.31 ± 0.33
Clinical attachment level (in mm)	2.62 ± 0.56*	2.70 ± 0.61
Periodontal screening index	3.18 ± 0.79*	3.10 ± 0.65
Highest degree of furcation involvement	0.55 ± 0.88*	0.68 ± 0.87
Number of teeth with furcation involvement	1.86 ± 3.49*	2.94 ± 5.12
Highest degree of tooth mobility	0.78 ± 0.39*	0.13 ± 0.34
Number of mobile teeth	0.14 ± 0.69*	0.55 ± 1.93
Bleeding on probing (in %)	21.58 ± 13.24*	17.53 ± 11.63

*No statistically significant differences compared to the control group (p > 0.05).

All assessed periodontal parameters were not significantly different between the groups. The Marfan group showed almost similar readings compared to the control group such as mean PPDs (2.33 ± 0.43 mm vs. 2.39 ± 0.40 mm) as well as mean GR readings (0.33 ± 0.34 mm vs. 0.31 ± 0.33 mm), mean CAL (2.62 ± 0.56 mm vs. 2.70 ± 0.61 mm), PSI (3.18 ± 0.79 vs. 3.1 ± 0.65), mean number of mobile teeth (0.14 ± 0.69 vs. 0.55 ± 1.93), and mean number of teeth with furcation involvement (1.86 ± 3.49 vs. 2.94 ± 5.12). The inflammation index BOP showed only a slight tendency to be higher in the Marfan group (21.58 ± 13.24% vs. 17.53 ± 11.63%).

### Dental examination

DMF-T and DMF-S were not significantly different between groups. Mean and range values are given in Table 2.

**Table 2:** Mean and range values of DMF-T and DMF-S and comparison of mean values (unpaired t-Test)

**Clinical**	**Marfan group**	**Control group**
**parameter**	**(n = 51)**	**(n = 31)**
DMF-T	14.55 ± 7.26*	13.71 ± 6.84
DMF-S	45.45 ± 31.34*	42.55 ± 32.16

*No statistically significant differences compared to the control group (p > 0.05).

## Discussion

Systemic manifestations and clinical characteristics of patients with MFS are well described by various authors. Data about oral manifestations of MFS such as maxillary protrusion, high palate, narrowed arch, crowding of the teeth, and fragility of the temporomandibular joint are consistent [8,9]. However, to date there is no data available in the dental/medical literature demonstrating a higher prevalence of periodontitis or more severe types of periodontitis in Marfan patients. Overall, various authors documented a higher prevalence of periodontitis in Marfan patients [12,21,22]. These studies always refer to reports published by *Straub et al.*[11] or *De Coster et al.*[10]. However it needs to be considered that *Straub et al.* reported only one case of severe periodontitis in MFS and *De Coster et al.* did not analyse the prevalence of periodontitis but only gingival inflammations. Therefore, the present study analysed for the first time the association of MFS with the prevalence and severity of periodontitis with an all-time sample size.

Firstly, our data do not indicate more periodontal damage, a higher prevalence or more severe forms of periodontitis in patients with MFS. Mean CAL and PSI were almost similar in the Marfan group compared to the sound controls. Several case reports [11-13] presented Marfan patients with gingival or periodontal abnormalities. In particular, *Straub et al.*[11] documented one case of MFS with severe periodontitis. But it must be kept in mind that the cause of attachment loss is multifactorial. Beside patient-specific risk factors like smoking, oral hygiene, nutrition, obesity, etc. there are patients with a genetic predisposition, which is up to now not well understood [23-26]. For example, ethnical origin may be an important risk factor. Africans suffer more frequently from so called aggressive forms of periodontitis [24]. Therefore, it may be concluded that a genetic predisposition and/or patient-specific periodontal risk factors independent from MFS may have been responsible for the severe periodontitis in the patient presented by *Straub et al.*[11].

Secondly, our data do not reveal significant differences rather than a slight tendency of more periodontal inflammation in Marfan patients compared to the control group. *De Coster et al*. [10] investigated 23 Marfan patients and compared dental and gingival conditions with 69 sound control patients. According to the results of the present study, *De Coster et al.* revealed more periodontal inflammation in Marfan patients compared to the control group (Gingiva Index in the Marfan group: 1.9 vs. 1.0 in the control group). However, *De Coster et al.* used a less reproducible index called Gingiva Index, which only assesses gingival inflammation signs by inspection. While interpreting the higher degree of inflammation, one has to keep in mind that Marfan patients suffer more frequently from a crowding of the teeth compared to sound persons. Crowded teeth are much more difficult to clean during patient’s oral hygiene. Thus, plaque accumulation and consequently periodontal inflammation is much more pronounced. Therefore, the higher degree of inflammation in patients with MFS may have been the result of crowded teeth.

Thirdly, the results of the dental examination revealed no elevated numbers of decayed, missing or filled teeth in the Marfan group compared to the control group. DMF-T and DMF-S were almost identical in both groups. This is confirmed by *De Coster et al.*[10], who also did not find significant differences between the mean DMF-T in 23 Marfan patients compared to 69 controls.

The analysis of the questionnaire revealed a relatively high prevalence of disorders of the temporomandibular joint, which is in accordance with other studies [8,9,27,28]. For instance, *Bauss et al.*[9] documented that 51.6% of 281 Marfan patients suffered from disorders of the temporomandibular joint. *Westling et al.*[8] reported a prevalence of 54% in 76 Marfan patients. Therefore, the temporomandibular joint is one of the main maxillofacial structures that are affected by the MFS. It may be concluded that orthodontic or skeletal anomalies like maxillary protrusion, high palate, or crowded teeth are responsible for the relatively high frequency of orthodontic treatment in Marfan patients due to our study (62%) and previous investigations [8,19]. *De Coster et al.*[10] analysed kephalometric data in 26 Marfan patients. They concluded based on their data that the need for orthodontic treatment in patients with MFS is significantly higher to sound controls.

Although the present study did not demonstrate a higher susceptibility to periodontitis and revealed only a slight tendency of more periodontal inflammation in patients with MFS one has to consider the increased risk for developing endocarditis in Marfan patients [6]. The higher risk for endocarditis is not the result of the disease but the consequence of a prosthetic valve or other prosthetic materials that are implanted in patients with MFS in order to treat cardiovascular complications. Therefore, an adequate dental monitoring and regular professional cleaning are recommended because of the higher number of patients with prosthetic valves suffering from Marfan syndrome.

## Conclusion

Based on our data, patients with Marfan syndrome do not suffer from more decayed, missing or filled teeth. The prevalence of disorders of the temporomandibular joint seems to be higher in Marfan patients then in patients without MFS. MFS patients do not reveal more periodontal damage, a higher prevalence of periodontitis or suffer more frequently from severe forms of periodontitis (e.g. aggressive periodontitis). It may be concluded that the mutation associated with MFS does not predispose the periodontal ligament to a higher susceptibility to periodontitis. Further, crowded teeth may be the reason for a slight tendency towards more periodontal inflammation in Marfan patients. Taken together, it is recommended that Marfan patients should receive a professional oral hygiene on a regular basis due to their increased risk of endocarditis based on the high number of Marfan patients with prosthetic heart valves.

## Competing interests

The authors declare that they have no competing interests.

## Authors’ contributions

IS performed the clinical examination, interpreted data and drafted the manuscript. CH documented and analyzed data. YvK and MB acquired patients with Marfan syndrome. AR, WG and OB planned the present case control study. AR performed the clinical examination. WG critically revised the manuscript. All authors read and approved the final manuscript.

## Pre-publication history

The pre-publication history for this paper can be accessed here:

http://www.biomedcentral.com/1472-6831/13/59/prepub
